# Muscleblind-like 1 antisense RNA 1 inhibits cell proliferation, invasion, and migration of prostate cancer by sponging miR-181a-5p and regulating PTEN/PI3K/AKT/mTOR signaling

**DOI:** 10.1080/21655979.2021.1890383

**Published:** 2021-03-01

**Authors:** Xiang Ding, Xu Xu, Xue-Feng He, Ye Yuan, Chuang Chen, Xin-Yu Shen, Sai Su, Zhang Chen, Song-Tao Xu, Yu-Hua Huang

**Affiliations:** a Department of Urology, The First Affiliated Hospital of Soochow University, Suzhou, China; b Department of Clinical Medicine, Luohe Medical College, Luohe, China

**Keywords:** Prostate cancer, MBNL1-AS1, invasion, migration, PTEN

## Abstract

The present study aimed to investigate the role and underlying mechanisms of long non-coding RNA (lncRNA) muscleblind-like 1 antisense RNA 1 (MBNL1-AS1) in the progression of Prostate cancer (PCa). MBNL1-AS1 and microRNA (miR)-181a-5p expression in PCa tissues and several human PCa cell lines were analyzed, respectively, using StarBasev3.0 project and RT-qPCR assay. After MBNL1-AS1 overexpression, cell proliferation, invasion and migration were, respectively, evaluated using CCK-8, colony formation, transwell and wound healing assays. Dual luciferase assay were used for analysis of the interactions among MBNL1-AS1, miR-181a-5p, and phosphatase and tensin homolog (PTEN). Subsequently, the expression of PTEN and proteins in PI3K/AKT/mTOR signaling was examined using western blot analysis after transfection with miR-181a-5p mimic. The rescue assays were performed to investigate the effects of MBNL1-AS1 and miR-181a-5p on the functions of PCa cells and the expression of PTEN/PI3K/AKT/mTOR signaling by co-transfection with MBNL1-AS1 plasmid and miR-181a-5p mimic. Results indicated that MBNL1-AS1 was conspicuously downregulated while miR-181a-5p upregulating in PCa tissues and cell lines. MBNL1-AS1 overexpression decreased the abilities of cell proliferation, invasion, and migration. Further study revealed that MBNL1-AS1 acted as a sponge for miR-181a-5p and positively regulated PTEN by a sponge effect. Additionally, rescue assays proved that the effect of MBNL1-AS1-upregulation on the proliferation, invasion, and migration of PCa cells was dependent on miR-181a-5p. Furthermore, miR-181a-5p overexpression counteracted the expression of PTEN and proteins in PI3K/AKT/mTOR signaling exerted by MBNL1-AS1-upregulation in PCa cells. This study suggests that MBNL1-AS1 inhibits the progression of PCa via sponging miR-181a-5p and regulating PTEN/PI3K/AKT/mTOR pathway.

## GRAPHICAL ABSTRACT

**Figure:**
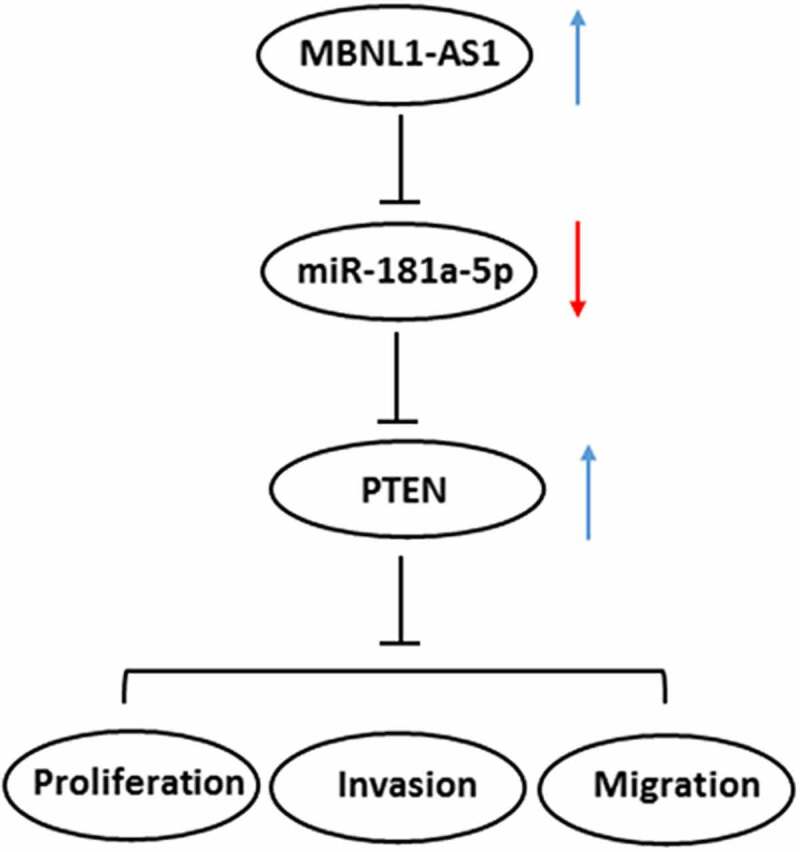

## Introduction

Prostate cancer (PCa) is one of the most common causes of cancer death all over the world threatening men’s health, which results in approximately three hundred thousand deaths every year [1,2]. Despite great progression of therapy methods in recent decades, the high rate of secondary metastasis still in poor prognosis. The exact internal mechanism remains difficult to define [3]. Therefore, it is urgent to obtain a comprehensive understanding of the molecular mechanisms involved in the development and progression of PCa to develop the more effective therapeutic target for the treatment of this disease.

Long noncoding RNAs (lncRNAs) are recognized as a class of non-coding RNAs with the length longer than 200 nucleotides, and do not exhibit any capacity to encode proteins [4]. A considerable body of evidence indicates that the expression of lncRNA is precisely regulated under physiological conditions and abnormal expression of it results in the pathogenesis of a wide range of diseases including cancers [5–7]. Substantial evidence exists to suggest that lncRNA dysregulation attributes a tumor-suppressor or an oncogenic role to lncRNAs affecting the clinicopathological appearance and prognosis in the PCa [8–10]. LncRNA muscleblind-like 1 antisense RNA 1 (MBNL1-AS1) is a newly discovered lncRNA in recent years. Based on emerging research, downregulation of MBNL1-AS1 contributes to tumorigenesis of non-small cell lung cancer by promoting cell proliferation, invasion, and migration via sponging miR-135a-5p [11]. Emerging evidence supports that MBNL1-AS1 inhibits cell proliferation and intensifies cell apoptosis in bladder cancer [12]. However, the roles of MBNL1-AS1 in the progression of PCa remain to be elucidated. By using StarBase v3.0 project, we found that MBNL1-AS1 expression was significantly decreased in tissue samples of patients with PCa. Therefore, we assess the effect of MBNL1-AS1 on the progression of PCa and identify the potential mechanisms.

MicroRNA (miRNA) are RNAs with no protein-coding abilities, and they have been demonstrated to be featured prominently in regulating the biological processes, such as proliferation, migration, and differentiation of cells [13,14]. Reportedly, in PCa, a large range of miRNAs are pivotal tumor promoters or tumor suppressors [15–17]. Emerging evidence indicated that miR-181a is identified as an oncogenic role in prostate tumor samples and prostate cancer cells [18]. By using bioinformatics analysis, Shen et al demonstrated that miR-181a is downregulated in PCa, and miR-181a is remarkably involved in regulating cell metabolic process and gene expression [19].

In this study, the expression of MBNL1-AS1 in several human PCa cell lines was detected firstly. After MBNL1-AS1 overexpression, the biological function of MBNL1-AS1 in PCa and MBNL1-AS1 mechanistically served as a competitive endogenous RNA (ceRNA) to regulate miR-181a-5p and phosphatase and tensin homolog (PTEN) were investigated. Findings in the current study may uncover a novel diagnostic and therapeutic target for the intervention of PCa.

## Materials and methods

### Cell culture

Several human PCa cell lines including LAPC4, LNCaP, DU145, and C4-2B and the normal prostate epithelial cell line RWPE-1 were obtained from the American Type Culture Collection (Manassas, VA, USA). Cells were incubated in RPMI 1640 medium (GIBCO, Grand Island, NY, USA) supplemented with 10% fetal bovine serum (Hyclone, Logan, UT, USA) in an atmosphere of 95% air and 5% carbon dioxide at 37 °C.

### Cell transfection

The MBNL1-AS1 overexpression plasmid (pc-MBNL1-AS1) and empty vector plasmid (pcDNA3.1) were the products of Shanghai GenePharma Co., Ltd. miR-181a-5p mimics and scrambled mimic negative control (miR-NC) were provided by RiboBio (Guangzhou, China). The LNCaP cells were transfected by using Lipofectamine 3000® (Invitrogen; Thermo Fisher Scientific, Inc.) as the carrier, according to the instructions of the kit. Successful transfection was verified using reverse transcription-quantitative PCR (RT-qPCR) analysis after 48 h of transfection.

### Cell viability assay

Cell viability was tested with a Cell Counting Kit-8 (CCK-8) kit purchased from Shanghai Yi Sheng Biotechnology Co. Ltd. (Shanghai, Chian). After transfection, LNCaP cells were seeded into 96-well plates with the density of 3 × 10^3^ cells per well. At each of the desired time points, plates were cultured for 4 h after each well was added with 10 μL CCK-8 solution. The optical density was detected at 450 nm using a microplate reader (Bio-Rad Laboratories, Inc., Hercules, CA, USA).

### Colony formation assay

For colony formation assay, LNCaP cells in the logarithmic growth phase were seeded in 6-well plates (500 cells/well) and were maintained for 14 days at 37°C until colony formation became visible. Cell colonies were washed with phosphate buffer solution (PBS), fixed with 4% paraformaldehyde, and stained with 0.5% crystal violet. Images were captured under an inversion microscope (Olympus Corporation; magnification, 10x). The number of colonies was counted by means of Image J software (version 1.52 r; National Institutes of Health).

### Transwell invasion assay

Cell invasion assay was performed using an 8-μm pore insert precoated with Matrigel (BD Biosciences). Transfected LNCaP cells (2x10^5^ cells) were resuspended in 200 µL serum-free RPMI 1640 medium and seeded on the upper chambers of the Transwell filters, and the lower chamber was filled with 600 μL of medium containing 10% FBS as a chemoattractant. After a 24-h incubation, cells invading the lower chamber were fixed with 4% formaldehyde, followed by stained with 0.1% crystal violet. Images were captured with an inverted light microscope (Olympus Corporation) at a magnification of x200. The numbers of invaded cells were counted from five randomized fields for detecting the cell invasion ability using Image J software (version 1.52 r; National Institutes of Health).

### Wound healing assay

Cell migration ability was evaluated by means of wound scratch healing assay. In brief, LNCaP cells (4x10^5^ cells/well) were transferred to 6-well plates and maintained in RPMI 1640 medium with 10% FBS. When the cell confluence reached ≥80%, a uniform straight wound was made by scraping the cell layer across each culture plate using a sterile pipette tip. Then, the floating debris were washed with PBS and the media was replaced with serum-free medium. After incubation for 24 h, the average distance of cells migrated into wound surface was captured under an inverted microscope (Olympus Corporation; magnification, 100 x). Quantitative analysis of the wound healing area was performed using Image J software (version 1.52 r; National Institutes of Health).

### Luciferase activity reporter assay

The StarBase database 3.0 (http://starbase.sysu.edu.cn/) was used to predict the associations between MBNL1-AS1 and miR-181a-5p, as well as miR-181a-5p and PTEN, which were validated with the Dual-Luciferase Reporter Assay System (Promega, Madison, WI). Cells were seeded into 24-well plates and incubated for 24 h before transfection. Subsequently, cells were co-transfected with pmirGLO plasmids (Promega) containing wild-type (WT) or mutant-type (Mut) MBNL1-AS1 or PTEN (all from Shanghai GenePharma Co., Ltd.), along with miR-181a-5p mimic or miR-NC using Lipofectamine 3000® reagent (Invitrogen; Thermo Fisher Scientific, Inc.). Based on manufacturer’s instructions, luciferase assays were performed after 48 h of incubation. Luciferase data were presented as Firefly luciferase activity normalized to the Renilla luciferase.

### RNA-binding protein immunoprecipitation (RIP) assay

The RIP assay was conducted with the RNA-Binding Protein Immunoprecipitation kit (Sigma-Aldrich; Merck KGaA) in accordance with the manufacturer’s guidelines. Briefly, LNCaP cells were collected and lysed through scraping cells in RIP lysis buffer. The cell lysates were incubated with magnetic beads conjugated with Argonaute-2 (anti-Ago2, Millipore) antibodies or Immunoglobulin G (anti-IgG, Millipore) at 4°C overnight. Normal IgG was the negative control. Subsequently, proteins were removed from the beads by incubation with proteinase K. The enrichment of PTEN was detected by RT-qPCR.

### RNA-pull down assay

RNA pull-down assay was conducted to determine the interaction between miR-181a-5p and PTEN with RNA-pull down assay using PierceTM Magnetic RNA-Protein Pull-Down Kit (Millipore, Billerica, MA, USA). Biotinylated PTEN or negative control probes were conjugated with streptavidin beads (Beyotime, Shanghai, China), which were transfected into LNCaP cells and incubated for 48 h. RT-qPCR estimated the purified RNA complex.

### RT-qPCR assay

The total RNAs from cells were extracted using Trizol agent (Takara, Tokyo, Japan). The complementary DNA (cDNA) was then synthesized by means of the SuperScript IV First-Strand Synthesis System (Invitrogen; Thermo Fisher Scientific, Inc.) following manufacturer’s recommendations. Subsequently, using cDNA as the template, the gene expression levels were analyzed by qPCR conducted using iTaq™ Universal One-Step iTaq™ Universal SYBR® Green Supermix (Bio-Rad Laboratories, Inc.) on an ABI 7500 instrument (Applied Biosystems; Thermo Fisher Scientific, Inc.). Glyceraldehyde 3-phosphate dehydrogenase (GAPDH) or U6 were used as internal controls for normalization. The 2^−ΔΔCq^ method was used to compare relative expression levels [20].

### Western blot analysis

The proteins were isolated from the treatment PCa cells by using RIPA buffer (Beyotime Institute of Biotechnology). The concentration of protein in each sample was evaluated with a bicinchonininc acid (BCA) Protein Assay kit (Beyotime Institute of Biotechnology). The protein samples (40 µg/lane) were subjected to 10% sodium dodecyl sulfate polyacrylamide gel electrophoresis (SDS-PAGE) and transferred onto polyvinylidene membranes (Millipore). Proteins were then blocked with 5% skimmed milk for 1.5 h, followed by incubated with specific primary antibodies at 4°C overnight. After washing three times with Tris Buffered saline (TBS)-0.2% Tween-20, these blots were incubated with a horseradish peroxidase-conjugated secondary antibody (Cell Signaling Technology, Inc.). Finally, the signals were visualized using an enhanced chemiluminescence assay (EMD Millipore). The grayscale values of the membranes were semi-quantified using an Image J software (version 1.52 r; National Institutes of Health). GAPDH was used as an internal control.

### Statistical analysis

Statistical analysis of the data was performed using GraphPad Prism 8.0 (GraphPad Software). All data were recorded as the mean ± standard deviation. The comparisons between two groups were analyzed by Student’s t-test. One-way analysis of variance (ANOVA) with Turkey’s post hoc test was used to compare the differences among groups. Two-way ANOVA was employed to conduct the differences in luciferase activity reporter assay. Differences with P < 0.05 were statistically significant.

## Results

### MBNL1-AS1 expression is markedly downregulated in PCa tissues and cell lines

**Figure 1.**
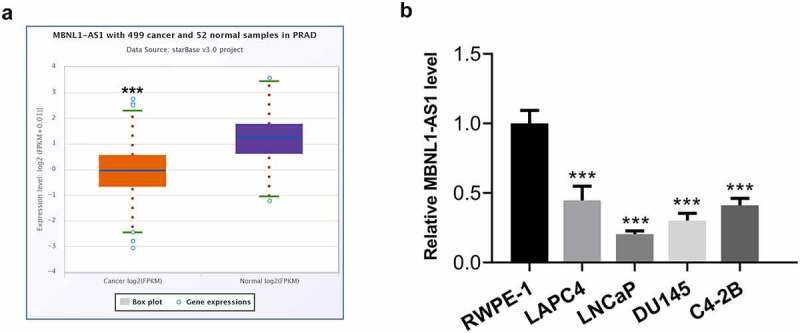
MBNL1-AS1 expression was remarkably downregulated in PCa tissues and cell lines. (a) The StarBase v3.0 project was used to analyze the level of MBNL1-AS1 in PCa tumor samples and normal samples. ***P < 0.001 vs. normal. (b) MBNL1-AS1 expression in several human PCa cell lines (LAPC4, LNCaP, DU145 and C4-2B) and the normal prostate epithelial cell line RWPE-1 was determined with RT-qPCR. ***P < 0.001 vs. RWPE-1

LncRNAs play significant roles in the progression of PCa. MBNL1-AS1 is a newly discovered lncRNA in recent years, which has been reported as a tumor suppressor in several cancers [11,12]. Firstly, the expression of MBNL1-AS1 in tissue samples of patients with PCa was analyzed by using StarBase v3.0 project. As what is observable from Figure 1a, MBNL1-AS1 expression was notably reduced in tissues of prostate adenocarcinoma (PARD) compared with the normal tissues. Additionally, significantly downregulated expression of MBNL1-AS1 was observed in human PCa cell lines, particularly in LNCaP cells, as a comparison to the normal prostate epithelial cell line RWPE-1 (Figure 1b). Therefore, the LNCaP cell line was selected for further experimentation, as it displayed the lowest MBNL1-AS1 expression level.

### MBNL1-AS1 overexpression represses the proliferation, invasion and migration of LNCaP cells

**Figure 2.**
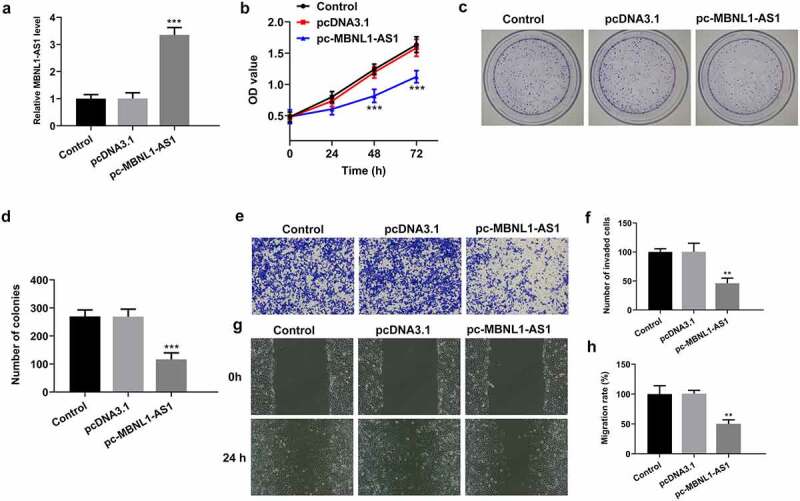
MBNL1-AS1 overexpression suppressed the proliferation, invasion and migration of LNCaP cells. (a) The expression of MBNL1-AS1 was examined using RT-qPCR after transfection with MBNL1-AS1 plasmid. ***P < 0.001 vs. pcDNA3.1. (b) Cell viability was evaluated by a CCK-8 kit. (c-d) The capacity of cell proliferation was assessed by means of colony formation assay. (e-f) Invasive activity of LNCaP cells was determined with Transwell assay. Magnification, x100. (g-h) Representative images and relative quantification of cell migration, as detected by a wound healing assay. Magnification, x100. **P < 0.01, ***P < 0.001 vs. pcDNA3.1

Due to the significant downregulation of MBNL1-AS1 expression in PCa tissues and cell lines, the possible biological functions of MBNL1-AS1 in the progression of PCa was investigated. MBNL1-AS1 was overexpressed by transfection with MBNL1-AS1 overexpression plasmid and this result was shown in Figure 2a. Then, it was found that MBNL1-AS1-upregulation conspicuously inhibited the viability of LNCaP cells compared with the empty vector group (Figure 2b). As what is observable from Figure 2c-D, the number of colonies was dramatically decreased after MBNL1-AS1 overexpression relative to the pcDNA3.1 group. Consistently, results of Transwell and wound healing assays displayed, respectively, in Figure 2e-h indicated that MBNL1-AS1 overexpression noticeably suppressed the abilities of LNCaP cell invasion and migration as comparison to the vector control group. These data suggest that overexpression of MBNL1-AS1 could inhibit the progression of PCa.

### MBNL1-AS1 is directly targeted by miR-181a-5p

**Figure 3.**
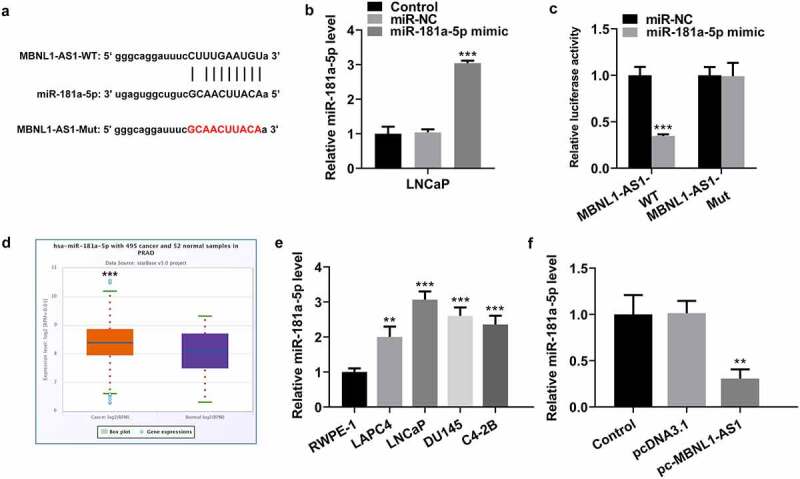
MBNL1-AS1 may bind to miR-181a-5p. (a) Potential binding sites of MBNL1-AS1 and miR-181a-5p. (b) The expression of miR-181a-5p was tested with RT-qPCR after transfection with miR-181a-5p mimic. ***P < 0.001 vs. miR-NC. (c) A luciferase activity reporter assay was conducted to verify the binding between MBNL1-AS1 and miR-181a-5p. ***P < 0.001 vs. miR-NC. (d) MiR-181a-5p level in PCa cancer samples and normal samples obtained from StarBase v3.0 project. ***P < 0.001 vs. normal. (e) RT-qPCR was employed to determine the expression of miR-181a-5p in several human PCa cell lines (LAPC4, LNCaP, DU145 and C4-2B) and the normal prostate epithelial cell line RWPE-1. ***P < 0.001 vs. RWPE-1. (f) MiR-181a-5p expression in LNCaP cells was evaluated by means of RT-qPCR analysis. **P < 0.01 vs. pcDNA3.1

To illustrate the potential molecular mechanism of MBNL1-AS1 in PCA, we predicted the potential target of MBNL1-AS1 using StarBase database 3.0 bioinformatics analysis. It was found that miR-181a-5p was the putative target of MBNL1-AS1, and the potential complementary binding sites was exhibited in Figure 3a. After miR-181a-5p overexpression (Figure 3b), a dual-luciferase reporter assay was performed to verify the combination between MBNL1-AS1 and miR-181a-5p. As presented in Figure 3c, transfection of miR-181a-5p mimic markedly reduced the luciferase intensity of MBNL1-AS1-WT in LNCaP cells, while the miR-181a-5p mimic did not exert an inhibitory effect on MBNL1-AS1-Mut. Additionally, significantly upregulated expression of miR-181a-5p was noticed in tissue samples of patients with PCa and several human PCa cell lines (Figure 3d,e). Subsequently, MBNL1-AS1 overexpression conspicuously repressed the expression of miR-181a-5p in LNCaP cells as comparison to the empty vector group (figure 3f). In collection, these results suggest that MBNL1-AS1 may bind directly to miR-181a-5p.

### PTEN is a direct target of miR-181a-5p

**Figure 4.**
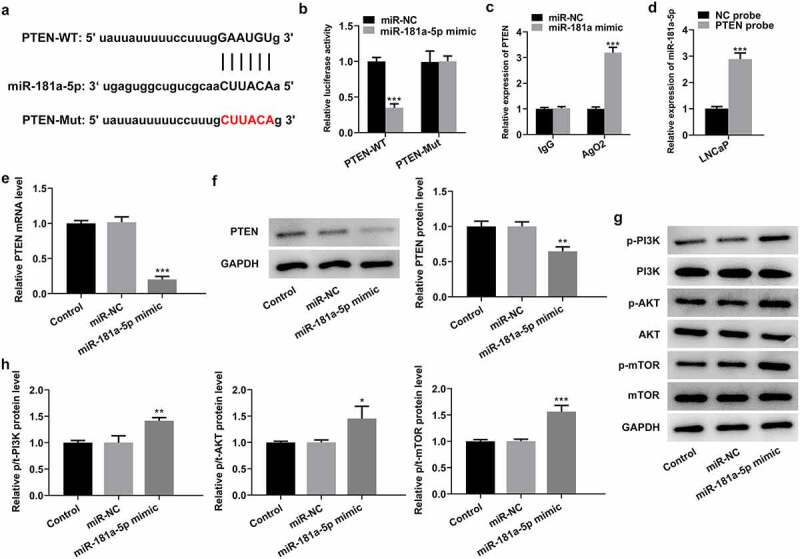
PTEN is a direct target of miR-181a-5p. (a) Potential binding sites of miR-181a-5p and PTEN. (b) Luciferase gene reporter assay displayed the activity when co-transfected with the PTEN-WT or Mut sequences and miR-181a-5p mimic or miR-NC. ***P < 0.001 vs. miR-NC. (c) RIP assay was used to verify the interaction between miR-181a-5p and PTEN. ***P < 0.001 vs. miR-NC. (d) RNA pull-down assay was employed to confirm the binding ability between miR-181a-5p and PTEN. ***P < 0.001 vs. NC probe. (e-f) Expression of PTEN was determined with RT-qPCR and western blot analysis after miR-181a-5p overexpression. (g-h) Western blotting was applied for the assessment of p-PI3K, p-AKT and p-mTOR following transfection with miR-181a-5p mimic. *P < 0.05, **P < 0.01 and ***P < 0.001 vs. miR-NC

To further investigate the regulatory mechanisms of MBNL1-AS1 and miR-181a-5p in PCa, the genes that potentially regulated by miR-181a-5p were analyzed by means of StarBase database 3.0 bioinformatics analysis. PTEN was found as a potential candidate, and the potential binding site of miR-181a-5p in the 3′UTR of PTEN is shown in Figure 4a. As what is observable from Figure 4b, the luciferase activity was significantly decreased following transfection with miR-181a-5p mimic and PTEN-WT, compared with that in cells co-transfected with miR-NC and PTEN-WT. Additionally, RIP experiment and RNA pull-down assay proved that PTEN could directly interact with miR-181a-5p (Figure 4c-d). Moreover, PTEN expression was evaluated with RT-qPCR and western blotting, respectively. It was found that miR-181a-5p mimic remarkably restrained the level of PTEN mRNA and protein compared with the miR-NC group (Figure 4e-f), supporting miR-181a-5p targets PTEN. Of note, notably intensified expression of p-PI3K, p-AKT, and p-mTOR was observed when miR-181a-5p was overexpressed by transfection with miR-181a-5p mimic (Figure 4g-h). To sum up, these observations reveal that PTEN is a direct target of miR-181a-5p.

### MiR-181a-5p mimic partially counteracts the inhibitory effects of MBNL1-AS1 overexpression on the proliferation, invasion, and migration of LNCaP cells

**Figure 5.**
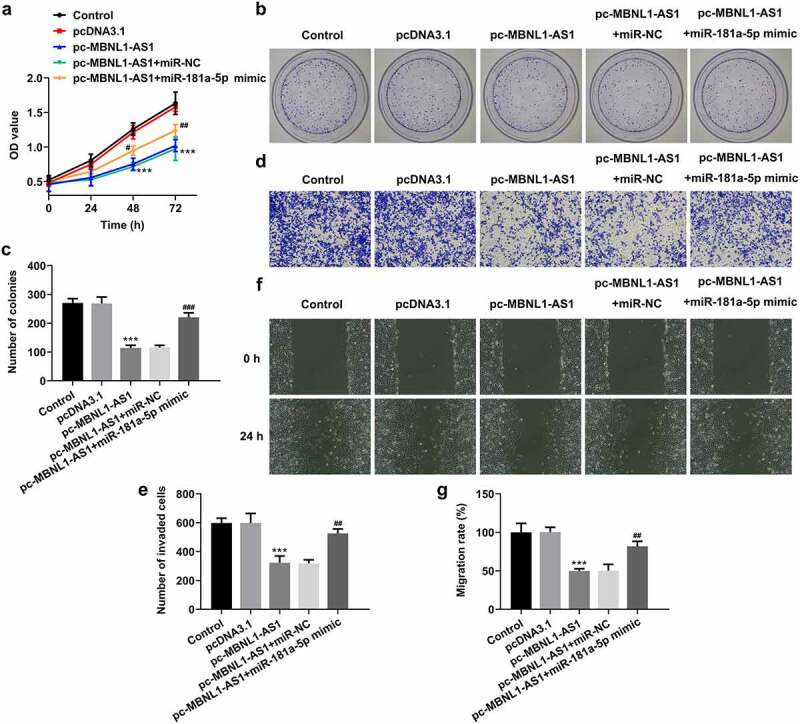
MiR-181a-5p mimic partially reversed the impact of MBNL1-AS1-upregulation on the proliferation, invasion and migration of LNCaP cells. (a) Cell viability was evaluated using CCK-8 assay. (b-c) Cell proliferation was detected by means of colony formation assay. (d-e) Transwell invasion assay was tested and the results were expressed as the number of invaded cells per field. (f-g) Wound scratch healing assay illustrated the migratory ability of LNCaP cells. ***P < 0.001 vs. pcDNA3.1; ^#^P < 0.05, ^##^P < 0.01 and ^###^P < 0.001 vs. pc-MBNL1-AS1+ miR-NC

Afterward, rescue experiments were performed to clarify the molecular mechanisms. As exhibited in Figure 5a, miR-181a-5p overexpression dramatically elevated the viability of LNCaP cells compared with pc-MBNL1-AS1+ miR-NC group. Simultaneously, it was found that the capacities of cell proliferation, invasion, and migration examined, respectively, by colonies formation (Figure 5b-c), Transwell (Figure 5d-e), and wound healing assays (figure 5f-g), were partially enhanced following co-transfection with pc-MBNL1-AS1 and miR-181a-5p mimic. Through the above findings, we proved that the effects of MBNL1-AS1 on the proliferation, invasion and migration of PCa cells are dependent on miR-181a-5p.

### MBNL1-AS1 activates PTEN to inhibit the PI3K/AKT/mTOR signaling pathway and miR-181a-5p reverses the MBNL1-AS1 effects in LNCaP cells

**Figure 6.**
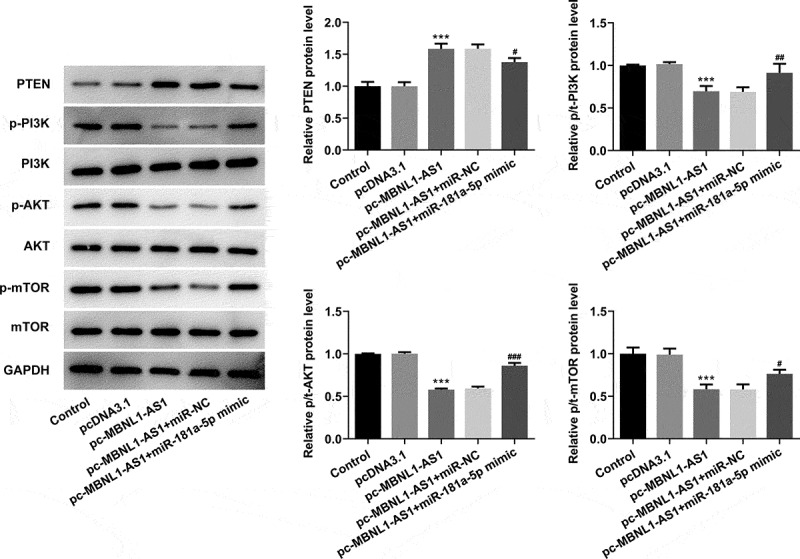
Upregulation of miR-181a-5p and MBNL1-AS1 attenuated PTEN expression and activates the PI3K/AKT/mTOR signaling pathway in LNCaP cells. The expression of PTEN, p-PI3K, p-AKT and p-mTOR was determined using western blot analysis. ***P < 0.001 vs. pcDNA3.1; ^#^P < 0.05, ^##^P < 0.01 and ^###^P < 0.001 vs. pc-MBNL1-AS1+ miR-NC

Then, the levels of PTEN and proteins in PI3K/AKT/mTOR signaling were evaluated using western blot analysis. As exhibited in Figure 6, MBNL1-AS1 overexpression significantly elevated the PTEN expression but reduced p-PI3K, p-AKT, and p-mTOR expression compared with the vector control group. By contrast, co-transfection with pc-MBNL1-AS1 and miR-181a-5p mimic remarkably downregulated the expression of PTEN while upregulating that of p-PI3K, p-AKT, and p-mTOR, as comparison to pc-MBNL1-AS1+ miR-NC group. Together, these findings provide evidence that miR-181a-5p overexpression notably counteracts the expression of PTEN and proteins in PI3K/AKT/mTOR signaling exerted by MBNL1-AS1-upregulation in PCa cells.

## Discussion

Continuing advances in the development of next-generation sequencing and transcriptomics suggest that the investigation of lncRNAs in the regulation of cancer has emerged as a potential research field [21]. Accumulating evidence shows that lncRNAs are involved in tumorigenesis and have the potential to be used as diagnostic and prognostic biomarker in a wide range of cancers [22,23]. Worldwide research has focused on identifying potential diagnostic and prognostic markers, which might predict survival outcomes accurately. The major finding of our work is that MBNL1-AS1 significantly downregulates in PCa tissues and cells, and MBNL1-AS1 inhibits the proliferation, invasion, and migration of PCa cells by sponging miR-181a-5p and regulating PTEN/PI3K/AKT/mTOR signaling pathway.

MBNL1-AS1, a novel found lncRNA located at the site of 3q25.1 in recent years, is first reported to be a differentially expressed lncRNA implicated in ischemia-reperfusion injury after total knee arthroplasty [24]. MBNL1-AS1 has been elucidated to be downregulated in non-small cell lung cancer, whose upregulation inhibits proliferation, invasion, migration, drug resistance, and sphere formation of cancer cells [25]. Additionally, Wei et al. demonstrate that MBNL1-AS1 acts as a tumor suppressor of bladder cancer via suppressing cell proliferation and promoting cell apoptosis through miR-135a-5p/PHLPP2/FOXO1 axis [12]. Emerging evidence supports that MBNL1-AS1 plays an inhibitory role in colon cancer by upregulating miR-412-3p-targeted MYL9 [26]. Here, we reported MBNL1-AS1 was notably decreased in PCa tissues and cells. An increase of MBNL1-AS1 repressed the progression of PCa, which was observed by the inhibition of cell proliferation, migration, and invasion.

Mechanically, a previous study has highlighted the importance of lncRNAs as competing endogenous RNAs (ceRNAs) in tumor biology [5]. It is known that lncRNAs function as ceRNAs to regulate tumor progression by sponging specific miRNAs, indirectly regulating gene expression at the post-transcriptional level [27,28]. MiRNAs are important in gene regulation and cellular processes in various diseases [29,30]. In our research, there was a binding site of miR-181a-5p in the sequence of MBNL1-AS1, and the dual-luciferase reporter assay confirmed that MBNL1-AS1 could directly target miR-181a-5p. In regard to the functions of miR-181a-5p in cancer, a considerable body of evidence indicates that miR-181a-5p exerts significant roles in tumorigenesis, such as multiple myeloma, endometrial carcinoma, and colorectal cancer [31–33]. Importantly, miR-181a is identified as an oncogenic role in prostate cancer cells and prostate tumor samples, and miR-181a promotes epithelial to mesenchymal transition of prostate cancer cells by targeting TGIF2 [18]. Moreover, migration and invasion inhibitory protein (MIIP) inhibits cell invasion and epithelial–mesenchymal transition in prostate cancer through miR-181a/b-5p-KLF17 axis [34]. Therefore, miR-181a is of great clinical significance in the treatment of PCa. In the current study, a series of functional experiments have indicated the regulatory association between MBNL1-AS1 and miR-181a-5p. Additionally, PTEN was confirmed as a potential target of miR-181a-5p in this study, which was in consistent with the previous studies [35,36]. PTEN, a tumor suppressor, is an antagonist regulator of the PI3K/AKT/mTOR signaling by inhibiting phosphorylation of PI3K, AKT, and mTOR, which was involved in the regulation of various cancers, including PCa, by participating in multiple tumor biological processes [37–39]. Intriguingly, the present study demonstrated that MBNL1-AS1 restrained the proliferation, invasion, and migration of PCa cells by sponging miR-181a-5p to activate PTEN and to further suppress the PI3K/AKT/mTOR signaling pathway.

## Conclusion

To conclude, our findings constitute the first report to delineate the biologic functions of MBNL1-AS1 in PCa. Results reveal the expression of MBNL1-AS1 is conspicuously downregulated in PCa tissues and cells. Overexpression of MBNL1-AS1 represses the proliferation, invasion, and migration of PCa cells via sponging miR-181a-5p and regulating the PTEN/PI3K/AKT/mTOR signaling pathway. These findings provide useful information to find new biomarkers for diagnosis and therapeutic application in the treatment of PCa. However, the use of only one PCa cell line in the cell function experiments and mechanism experiments is a limitation of the present study. Therefore, a comprehensive analysis is required in the future.

## Data Availability

All data generated or analyzed during this study are included in this published article.
